# Abnormal Visual Function: An Under-recognized Risk Factor of Road Traffic Injuries

**DOI:** 10.18502/jovr.v17i4.12306

**Published:** 2022-11-29

**Authors:** Hassan Hashemi, Payam Nabovati, Abbasali Yekta, Ali Borojerdi, Hamidreza Fallahkohan, Farhad Rezvan, Mehdi Khabazkhoob

**Affiliations:** ^1^Noor Research Center for Ophthalmic Epidemiology, Noor Eye Hospital, Tehran, Iran; ^2^Rehabilitation Research Center, Department of Optometry, School of Rehabilitation Sciences, Iran University of Medical Sciences, Tehran, Iran; ^3^Department of Optometry, Mashhad University of Medical Sciences, Mashhad, Iran; ^4^Budget and Planning Office of Road Maintenance and Transportation Organization, Tehran, Iran; ^5^Noor Ophthalmology Research Center, Noor Eye Hospital, Tehran, Iran; ^6^Department of Medical Surgical Nursing, School of Nursing and Midwifery, Shahid Beheshti University of Medical Sciences, Tehran, Iran; ^7^https://orcid.org/0000-0002-6086-1537

**Keywords:** Case–Control Study, Contrast Sensitivity, Road Traffic Injury, Visual Field

## Abstract

**Purpose:**

To determine the relationship between road accidents with visual acuity, refractive errors, visual field, and contrast sensitivity.

**Methods:**

This population-based case–control study was conducted on roads leading to Tehran Province, Iran. The case group comprised drivers who had met with accidents and were at fault for the accident. The cases were selected in an ongoing manner (incidence cases). The controls were drivers who were the opposing victims in the same. After an initial interview, optometric and ophthalmic examinations including the measurement of visual acuity, refraction, visual field assessment, contrast sensitivity measurement, and slit lamp biomicroscopy were performed for all study participants.

**Results:**

In this study, 281 and 204 individuals were selected for the case and control groups. The mean uncorrected visual acuity was 0.05 ± 0.12 and 0.037 ± 0.10 logMAR in the case and control groups, respectively (*P* = 0.095). Of the participants in the case and control groups, 32.8% and 23% had a visual field defect in at least one eye, respectively (adjusted odds ratios [aOR] = 1.63, 95% confidence interval [CI]: 1.08–2.48; *P* = 0.021). Moreover, 16.2% of the cases and 8.3% of the controls had visual field defects in both eyes (aOR = 2.13, 95% CI: 1.17–3.86; *P* = 0.012). Contrast sensitivity was worse in the case group in all spatial frequencies under non-glare conditions. However, under glare conditions, the contrast sensitivity was significantly worse in the case group only in the spatial frequency of 12 cycles per degree (cpd).

**Conclusion:**

Reduced contrast sensitivity, especially under non-glare conditions, and visual field defects are risk factors that influence the prevalence of road accidents. It is strongly advised that special attention be paid to these visual functions in legal assessments to apply the necessary interventions in individuals with these types of disorders.

##  INTRODUCTION

In 2018, the World Health Organization (WHO) reported an increase in the number of road accident fatalities to 1.35 million victims per year with 20–50 million people suffering from non-fatal injuries.^[1]^ Globally, road accidents are the eighth cause of death in all ages and the leading cause of death in children and adolescents aged 5–29 years.^[2]^ According to available reports, road traffic injuries are the second leading cause of mortality in Iran.^[3,4]^ Therefore, it is very important to identify the risk factors associated with road accidents to reduce mortality through necessary measures.^[5]^

Driving is a complex task which is affected by different sensory, mental, motor, and compensatory capabilities of human body, among which the visual system provides > 90% of the input information required for driving.^[6,7]^Therefore, the process of driving is considered a visually demanding task.^[8]^ Due to the importance of the visual system, multiple studies have evaluated the relationship between the visual system function and the driving process and safety.^[8]^ Several studies have pointed out the importance of including evaluations of varied aspects of the visual system function such as visual acuity,^[9,10,11]^ contrast sensitivity,^[12,13,14]^ color vision,^[15,16]^ and visual field^[17,18]^ in primary and periodic driving qualification examinations.

It should be mentioned that most of the studies in this regard were descriptive studies that have merely assessed the visual system status or the prevalence of visual disorders or ocular pathologies in a group of drivers. Therefore, due to the lack of a control group, it is not possible to judge on the relationship between these visual parameters and the increased chance of accidents with certainty. Moreover, many of these studies investigated the relationship between a limited number of visual indices and the drivers' safety and performance without controlling the effects of confounding factors. It should be noted that different parameters of the visual system affect each other.^[19]^ To comment on the association of each of these parameters with the drivers' safety and performance, it is necessary to evaluate them simultaneously and control their confounding effects on other parameters. Few studies included a control group; however, they reported inconsistent and even contradictory results regarding the association between different visual indices and car accidents, indicating the need for further research in this regard. In addition, the extent of the effect of each visual parameter on the drivers' performance may be different depending on the geographical conditions (weather, road lighting) of each country. The present study aimed to determine the visual risk factors associated with the occurrence of road car accidents. Identifying these visual risk factors can help reduce road accidents and their fatalities through appropriate periodic visual qualification examinations as well as necessary ophthalmic therapeutic measures and environmental modifications.

##  METHODS

This population-based case–control study was conducted on roads leading to Tehran Province from September 2018 to March 2019. The study population comprised drivers that drove on the roads leading to Tehran Province during the study period. The subjects were selected using convenience sampling.

In this study, exposure and outcome were defined as visual system disorders and being at fault for the accident, respectively. The drivers that were involved in accidents during the study period according to police reports and were recognized at fault for the accident were considered as the cases and selected in an ongoing or incidence case manner. The control group included drivers who were the opposing victims in same accidents and were not at fault. So, the controls were matched with cases in terms of confounding factors such as the road on which the accident occurred and the accident time. Since the road's physical status and driving time can significantly affect the odds of traffic accidents, it seemed necessary to select the controls among the drivers who matched with the cases in terms of these two factors. Sampling was continued until the required sample size was achieved in each group.

The subjects that were involved in accidents in the aforementioned roads were included in the study if the accident did not affect their level of consciousness. Exclusion criteria were a history of using drugs, alcohol, and psychedelics, an unwillingness to participate in the study, and any physical condition hampering the interview. The official police report of the accident was also used to collect the required data. Finally, all of the subjects were invited to participate in further interviews and examinations.

Informed consent was obtained from all participants before entering the study. Interviews were performed to collect demographic data, education level, history of driving, ocular diseases, type of driver's license, type of vehicle, type of accident, and extent of injury. In addition, a brief history of previous accidents, time of driver's license renewal, and examinations were also obtained. Subjects were referred for examination after the interview. The interviewer was unaware of the study objectives and was masked to the case and control groups.

All study participants underwent optometric examinations by two experienced optometrists who were unaware of the study objectives and were masked to the case and control groups. The agreement between the two examiners was > 85% for the measurement of uncorrected visual acuity and refraction. First, uncorrected distance visual acuity (UCVA) was measured at 6 m using a Snellen E chart. Then, objective refraction was performed using the Topcon AR-8800 auto-refractometer (Topcon Corp, Tokyo, Japan) and the results were refined using the Heine Beta 200 retinoscope (HEINE Optotechnic, Germany). Next, optimal distance optical correction was determined using subjective refraction and the best-corrected distance visual acuity (BCVA) was measured. Contrast sensitivity was measured at spatial frequencies of 3, 6, 12, and 18 cycles per degree (cpd) using the CVS-1000 test (VectorVision, Greenville, SC, USA). Contrast sensitivity was measured with the best optical correction for subjects with a UCVA of < 20/20. All participants underwent contrast sensitivity measurements (in log units) under non-glare and glare conditions at 2.5 m from the measuring apparatus. After contrast sensitivity testing, static perimetry was performed by an experienced optometrist using the SITA-Standard 24-2 protocol with a white target using the Humphrey visual field analyzer (Carl Zeiss, Dublin, CA, USA). Finally, all study participants underwent slit-lamp biomicroscopy by an ophthalmologist.

In this study, hyperopia and myopia were defined as a spherical equivalent (SE) of subjective refraction worse than –0.5 D and +0.5 D, respectively. A visual field defect was defined as any result outside the normal limits on the final printout. The area under the contrast sensitivity curve (AUC) was calculated based on the following formula:

AUC = 0.477 × mean (3 cpd, 6 cpd) + 0.7782 × mean (6cpd, 12cpd) + 0.7782 × mean (12 cpd, 18 cpd)

### Statistical Analysis

Considering visual impairment as the main outcome, its prevalence varies in individuals with car accidents versus normal population groups according to different studies.^[20]^ By considering 12% (P1) and 7% (P2) prevalence of visual impairment in the accident and non-accident groups (normal population),^[21,22]^ respectively, according to previous studies, a type 1 error (α) of 0.05, and power (β) of 0.80, 270 subjects were required in each group based on the following formula: 


$$n=\frac{{\left(z_{1-\frac{\alpha }{2}}+z_{1-\beta }\right)}^{2}\bar{p}\bar{q}}{{\left(p_{1}-p_{2}\right)}^{2}}=270.$$

Data were presented as frequency and percentage along with mean and standard deviation (SD). Binary logistic regression was applied to evaluate the association between visual parameters and accidents in the case and control groups. Finally, a multivariable logistic regression model was used to control the effects of the confounding factors, and the results of the final model, which was run in a backward-stepwise manner, were reported as crude odds ratios (cOR) or adjusted odds ratios (aOR).

### Ethical Considerations

The tenets of the Declaration of Helsinki were considered in all stages of the study. Informed consent was obtained from all participants.

##  RESULTS

Two hundred and thirty-six subjects were selected for the control group and 281 for the case group, of whom 204 individuals in the control group (86.4%) and 259 individuals in the case group (92.2%) participated in the study. The mean age of the participants was 40.66 ± 10.30 and 39.57 ± 9.78 years in the case and control groups, respectively (*P* = 0.258).

The mean UCVA was 0.05 ± 0.12 logMAR in the case group and 0.037 ± 0.10 logMAR in the control group (*P* = 0.095). An UCVA of equal to or worse than 20/40 in the better eye was found in 7.8% of the cases versus 3.9% of the controls (cOR = 2.05, 95% CI: 0.88–4.75); however, there was no statistically significant difference in terms of BCVA between the two groups (*P* = 0.854).

The mean SE was –0.68 ± 1.20 diopters (D) in the case group and –0.037 ± 1.04 D in the control group (*P* = 0.774). The prevalence of myopia was 27.3% and 24% and the prevalence of hyperopia was 25% and 29% in the case and control groups, respectively (*P* = 0.419 and *P* = 0.339). The results of myopia and hyperopia did not change after an adjustment for age. Astigmatism was found in 55.6% of the subjects in the case group and 57.4% of the individuals in the control group (*P* = 0.695).

The results showed that 32.8% and 23% of the participants in the case and control groups had a visual field defect in at least one eye, respectively (cOR = 1.63, 95% CI: 1.08–2.48; *P* = 0.021); this relationship was still significant after adjusting for the effects of age and visual acuity in logMAR (aOR = 1.58, 95% CI: 1.03–2.42; *P* = 0.035). Moreover, 16.2% of the cases and 8.3% of the controls had visual field defects in both eyes (cOR = 2.13, 95% CI: 1.17–3.86; *P* =. 0.012); this significant relationship was still observed after adjusting for the effects of age and visual acuity (aOR = 1.90, 95% CI: 1.04–3.49; *P* = 0.037).

Table 1 presents the mean contrast sensitivity values in log units in different spatial frequencies under glare and non-glare conditions in the case and control groups. The case group had worse contrast sensitivity in all spatial frequencies under non-glare conditions (*P* < 0.001); however, under the glare conditions, the contrast sensitivity was significantly worse in the case group only in the spatial frequency of 12 cpd. In addition, according to Table 1, the AUC of contrast sensitivity was significantly worse in the case group than in the control group in both glare and non-glare conditions. The relationship between contrast sensitivity in different spatial frequencies and being at fault for the accident was evaluated after adjusting for the effects of age and visual acuity. Under glare conditions, no significant relationship was found between contrast sensitivity in different spatial frequencies and being at fault in the accident; however, the AUC of contrast sensitivity was worse in the case group in a marginally significant manner (*P* = 0.089).

**Table 1:** Contrast sensitivity values in log units in the case and control groups.


	**Spatial frequency**	<statement> <title>Case </title> </statement>	**Control**
Glare	3 cpd	1.65 ± 0.17	1.68 ± 0.16
	6 cpd	1.79 ± 0.19	1.82 ± 0.17
	12 cpd	1.38 ± 0.25	1.43 ± 0.24
	18 cpd	0.91 ± 0.25	0.95 ± 0.23
	AUC	2.94 ± 0.40	3.02 ± 0.36
Non-glare	3 cpd	1.72 ± 0.18	1.76 ± 0.12
	6 cpd	1.86 ± 0.18	1.92 ± 0.16
	12 cpd	1.51 ± 0.23	1.57 ± 0.20
	18 cpd	1.02 ± 0.23	1.07 ± 0.20
	AUC	3.15 ± 0.38	3.25 ± 0.29

Cpd, cycles per degree; AUC, area under the curve			

**Table 2:** The association between contrast sensitivity in different spatial frequencies under glare and non-glare conditions and being at fault for the accident after adjusting for the effect of age and visual acuity in a multivariable logistic regression model.


	**Contrast sensitivity**	**Age**	**Uncorrected visual acuity**
	**Spatial frequency**	**aOR (95%CI); ** ***P*** **-value**	**aOR (95%CI); ** ***P*** **-value**	**aOR (95%CI); ** ***P*** **-value**
Glare	3 cpd	0.44 (0.13–1.50); 0.190	1.01 (0.99–1.03); 0.525	0.95 (0.86–1.05); 0.342
	6 cpd	0.56 (0.19–1.61); 0.279	1.01 (0.99–1.03); 0.472	0.95 (0.86–1.05); 0.343
	12 cpd	0.48 (0.21–1.09); 0.079	1.01 (0.99–1.03); 0.526	0.96 (0.87–1.07); 0.466
	18 cpd	0.58 (0.26–1.31); 0.191	1.01 (0.99–1.03); 0.451	0.96 (0.87–1.06); 0.384
	AUC	0.63 (0.37–1.07); 0.090	1.01 (0.99–1.03); 0.542	0.96 (0.87–1.07); 0.470
Non-glare	3 cpd	0.24 (0.06–0.93); 0.039	1.01 (0.99–1.03); 0.566	0.95 (0.86–1.05); 0.323
	6 cpd	0.20 (0.06–0.69); 0.011	1.00 (0.98–1.02); 0.695	0.96 (0.87–1.07); 0.473
	12 cpd	0.34 (0.13–0.85); 0.021	1.00 (0.99–1.02); 0.625	0.97 (0.88–1.07); 0.545
	18 cpd	0.46 (0.19–1.15); 0.096	1.01 (0.99–1.03); 0.540	0.96 (0.87–1.06); 0.418
	AUC	0.44 (0.24–0.82); 0.009	1.00 (0.98–1.02); 0.735	0.97 (0.88–1.08); 0.591

aOR, adjusted odds ratio; cpd, cycles per degree; AUC, area under the curve				

Contrast sensitivity values at different spatial frequencies were highly correlated with each other. Therefore, the relationship between contrast sensitivity in each spatial frequency with the case and control groups was evaluated separately in a backward multivariable logistic regression model by controlling the effects of age and visual acuity. The results of the backward multivariable logistic regression for each spatial frequency and the AUC are shown separately in Table 2. As seen, after controlling for the effects of age and visual acuity, the contrast sensitivity under non-glare conditions at spatial frequencies of 3, 6, and 12 and the AUC were significantly worse in the case group.

##  DISCUSSION

The current study showed no significant association between corrected visual acuity and the risk of road traffic injuries, which is consistent with most previous studies. A review of the relationship between visual acuity and risk of vehicle accidents in 1999 found that the relative risk values reported in previous studies were often < 2 with a few exceptions; therefore, the authors concluded that there was a weak association between reduced visual acuity and risk of road accidents.^[23]^ Keefe et al studied 2594 subjects and found no significant difference in the risk of car crashes between individuals with a visual acuity of < 6/12 compared to those with a better visual acuity.^[9]^ Several other studies have also indicated no significant association between visual acuity and the risk of car accidents.^[11,13,24,25]^ Several factors may explain this lack of significant relationship. The first may be low variation in the visual acuity of the participants, because most of the studies compared intermediate visual acuities with relatively normal or slightly lower visual acuities. Another factor may be that decreased visual acuity has an overt nature and any individual is usually aware of this problem. Therefore, drivers with compromised visual acuity may deliberately adopt a series of compensatory reactions like limiting driving, avoiding driving at night and in risky conditions, and avoiding high-risk behaviors while driving,^[26,27]^ resulting in decreased risk of car accidents.

The results of this study showed a significant relationship between reduced contrast sensitivity and increased risk of road accidents. Several previous studies revealed a relationship between contrast sensitivity disorders and increased risk of car accidents. Marottoli et al reported a twofold increase in the risk of car accidents in individuals with impaired contrast sensitivity compared to those with normal contrast sensitivity.^[12]^ Cross et al also studied 363 car crashes and concluded that a high percentage of these accidents were due to impairment in the contrast sensitivity of the drivers.^[13]^ In 2001, Owsley et al found that cataract and resulting decreased contrast sensitivity played significant roles in car crashes leading to disabling or death. According to the results of the present and previous studies, it can be concluded that the risk of car crashes is markedly higher in people with compromised contrast sensitivity, which indicates a direct correlation between contrast sensitivity disorders and risk of car crashes.^[14]^

Visual field was another risk factor for assessing the impact on the incidence of car accidents in the present study. The results showed that the prevalence of bilateral visual field defects was significantly higher in the case group as compared to the control group. There are contradictory reports of the relationship between visual field defects and the drivers' safety and performance in the literature. Primary studies did not show such a relationship while later studies proved this association. Danielson did not find a significant relationship between the horizontal visual field and risk of car crashes.^[28]^ Similarly, Adekoya et al also found no significant association between visual field defects and road traffic accidents.^[18]^ However, Johnson and Keltner studied 10,000 subjects and found that bilateral visual field defects significantly increased the risk of car accidents.^[29]^ On the other hand, Haymes et al showed a six-time increased risk of car accidents in subjects with glaucoma and visual field defects as compared to other drivers.^[30]^ In 2015, Huisingh et al concluded that severe bilateral visual field defects increased the risk of road traffic accidents by 40%. Moreover, they reported that the risk was higher if the defect was in the inferior or left visual field.^[31]^

A limitation of the present study is that there are various environmental, psychological, and mental factors that may affect the relationship between visual functions and road accidents. It seems impossible to control all these factors. Therefore, these potential confounders must also be considered when interpreting the study findings.

In summary, according to the findings of the present study, decreased contrast sensitivity and visual field defect were risk factors that influenced the prevalence of road accidents. Since these two parameters are not routinely assessed in the driver's physical qualification examination in Iran, it is strongly advised that special attention be paid to these visual functions in legal assessments to apply the necessary interventions in subjects with these disorders. Moreover, it is recommended that a series of environmental modifications be applied to reduce the effects of visual disorders on the performance of drivers.

##  Financial Support and Sponsorship 

This project was financially supported by Noor Research Center for Ophthalmic Epidemiology.

##  Conflicts of Interest: 

No conflicting relationship exists for any author.
